# PSMA-RLT in Patients with Metastatic Hormone-Sensitive Prostate Cancer: A Retrospective Study

**DOI:** 10.3390/cancers15010297

**Published:** 2022-12-31

**Authors:** Amina Banda, Bastiaan M. Privé, Youssra Allach, Maike J. M. Uijen, Steffie M. B. Peters, Cato C. Loeff, Martin Gotthardt, Constantijn H. J. Muselaers, J. Alfred Witjes, Inge M. van Oort, J. P. Michiel Sedelaar, Harm Westdorp, Niven Mehra, Fadi Khreish, Samer Ezziddin, Amir Sabet, Michael C. Kreissl, Thomas Winkens, Philipp Seifert, Marcel J. R. Janssen, Willemijn A. M. van Gemert, James Nagarajah

**Affiliations:** 1 Department of Radiology and Nuclear Medicine, Radboud University Medical Center, P.O. Box 9101, 6525 GA Nijmegen, The Netherlands; 2 Department of Medical Oncology, Radboud University Medical Center, 6500 HB Nijmegen, The Netherlands; 3 Department of Urology and Oncology, Radboud Universiteit Medical Center, 6525 GA Nijmegen, The Netherlands; 4 Department of Nuclear Medicine, University of Saarland, D-66421 Homburg, Germany; 5 University Hospital, Department of Nuclear Medicine, Goethe University Frankfurt, 60590 Frankfurt am Main, Germany; 6 Division of Nuclear Medicine, Department of Radiology and Nuclear Medicine, Magdeburg University Hospital, 39120 Magdeburg, Germany; 7 Clinic for Nuclear Medicine, University Hospital of Jena, 07743 Jena, Germany

**Keywords:** Actinium-225, Lutetium-177, early stage, prostate cancer, PSMA radioligand therapy

## Abstract

**Simple Summary:**

Prostate-specific membrane antigen-direct radioligand therapy is a novel treatment for patients with castration-resistant prostate cancer. Yet, given the mode of action of PSMA-RLT, it is postulated that in early disease prostate cancer settings, e.g. hormone-sensitive, can also benefit from this treatment. In this retrospective study, the safety and efficacy was investigated of two PSMA-RLT schemes: monotherapy with 177Lu-PSMA and 177Lu-PSMA in combination with 225Ac-PSMA in twenty patients with early stage metastatic prostate cancer. The treatment appeared safe with limited and mainly transient side effects, also on a longer term follow-up, with encouraging efficacy for twenty early-stage metastatic prostate cancer.

**Abstract:**

Background: Prostate-specific membrane antigen (PSMA)-directed radioligand therapy (RLT) is a novel treatment for patients with castration-resistant prostate cancer (CRPC). Given the mode of action, patients in an earlier disease stage, such as hormone-sensitive prostate cancer (HSPC), are also likely to benefit from [^177^Lu]Lu-PSMA- (^177^Lu-PSMA) or [^225^Ac]Ac-PSMA-radioligand treatment (^225^Ac-PSMA). In this retrospective study, we analyzed the safety and efficacy of PSMA-RLT in early-stage and hormone-sensitive metastatic prostate cancer patients. Methods: A retrospective study was performed in patients who received ^177^Lu-PSMA and/or ^225^Ac-PSMA with early-stage metastatic prostate cancer. The primary outcome parameter evaluated in this study was the progression-free survival (PFS) after PSMA-RLT and toxicity according to the Common Terminology Criteria for Adverse Events. Secondary outcome parameters were prostate-specific antigen (PSA) response and the date of onset of CRPC state. Results: In total, 20 patients were included of which 18 patients received ^177^Lu-PSMA radioligand and two patients received tandem treatment with both ^177^Lu-PSMA and ^225^Ac-PSMA radioligands. Patients received a median of 2 treatment cycles (range 1–6) and a median activity of 6.2 GBq ^177^Lu-PSMA per cycle (interquartile range (IQR) 5.2–7.4 GBq). PSMA-RLT was overall well-tolerated. The most common grade 1–2 side effects were xerostomia (*n* = 6) and fatigue (*n* = 8), which were only temporarily reported. One patient that received ^225^Ac-PSMA developed grade 3–4 bone marrow toxicity. The median PFS was 12 months (95% confidence interval (CI), 4.09–19.9 months). Seventeen (85%) patients had a ≥50% PSA response following PSMA-RLT. One patient developed CRPC 9 months following PSMA-RLT. Conclusions: In this small cohort study, PSMA-RLT appeared safe and showed encouraging efficacy for (metastasized) early-stage and hormone-sensitive prostate cancer patients. Prospective studies are awaited and should include long-term follow-up.

## 1. Introduction

Prostate cancer is the most common malignancy in males [1]. Patients with a metastatic disease generally receive androgen deprivation therapy (ADT) as a first-line treatment. Despite favorable response to ADT, this treatment modality is associated with significant treatment-related side effects such as flushes, loss of libido, depression, osteoporosis, and cardiovascular disease, which can significantly affect the quality of life [2]. Therefore, both patients and physicians are increasingly searching for alternative strategies to defer or delay ADT.

Prostate-specific membrane antigen (PSMA) radioligand therapy (RLT) labeled with the beta-emitting ^177^Lutetium is a novel treatment for metastatic prostate cancer, with pending registration in 3rd or 4th line metastatic castration-resistant prostate cancer (mCRPC) [3]. However, it is postulated that patients in an earlier disease setting can also benefit from PSMA-RLT, i.e., in the castration-resistant phase. Recently, we reported encouraging outcomes of a prospective phase I study in which ten early-stage patients with hormone-sensitive prostate cancer received two cycles of [^177^Lu]Lu-PSMA-617 radioligand treatment (^177^Lu-PSMA) [4,5]. At present, a following randomized phase II study in the same setting is recruiting patients [6,7]. Moreover, encouraging data was recently presented at the 2022 congress of the European Association of Urology of the LuTectomy trial which applied 177Lu-PSMA prior to the prostatectomy.

While worldwide registry and availability of ^177^Lu-PSMA radioligand therapy are yet awaited, several patients were allowed to receive ^177^Lu-PSMA or the alpha-emitting ^225^Ac-PSMA in either study or in a compassionate use setting. This resulted in several studies reporting encouraging results of ^177^Lu-PSMA and ^225^Ac-PSMA (or a combination of both, so-called ‘Tandem therapy’) in patients in the mCRPC disease stage [8,9,10,11,12,13,14]. Due to the promising outcomes, some individuals that had unacceptable toxicities from ADT or chemotherapy were also treated in an earlier disease setting, e.g., prior to the castration-resistant stage, or even before initiation hormonal therapy to postpone this treatment. At present, there is a lack of published clinical data regarding the use of PSMA-RLT prior to the castration-resistant stage. We retrospectively evaluated the safety and therapeutic response of patients who received ^177^Lu-PSMA and/or ^225^Ac-PSMA in hormone sensitive or early-stage metastatic prostate cancer, mostly prior to initiation of hormonal therapy.

## 2. Materials and Methods

### 2.1. Study Design and Cohort Population

Between 12 September 2016 and 1 June 2021, patients with early-stage metastatic prostate cancer, mostly prior to the initiation of hormonal therapy, were evaluated in a retrospective cohort study. Patients with proven CRPC were excluded from this study. All patients were registered in Radboud University medical center (Radboudumc) and treated with PSMA-RLT either locally or in a collaborating center. Patients were well-informed about the standard of care therapeutic options by their treating urologist, oncologist and nuclear medicine physician. However, patients were either ineligible to the standard of care or refused any other conventional treatment due to unacceptable side effects. After being thoroughly informed on PSMA-RLT and its experimental position in this early setting, they chose to have PSMA-RLT. To evaluate tumor PSMA expression, a PSMA positron-emission tomography/computed tomography (PET/CT) scan was acquired prior to PSMA-RLT. Furthermore, to have a legitimate treatment evaluation, a minimum of 6 weeks of follow-up was required. All consecutive RLT cycles were included if no new cancer therapeutic agent was initiated. No exclusions were made due to longer time intervals between the PSMA-PET/CT and PSMA-RLT. Ten of our patients were previously published by Privé et al., 2021.

Demographic data, histopathology, diagnostic parameters, (prior-)treatments, toxicity and follow-up data were collected and recorded in a Castor electronic case report form (https://www.castoredc.com/ (accessed on 14 June 2021)).

### 2.2. PSMA Imaging and Therapy

The labelled compounds [^68^Ga]Ga-PSMA-11 (^68^Ga-PSMA), [^18^F]F-PSMA-1007 (^18^F-PSMA), ^177^Lu-PSMA and ^225^Ac-PSMA were manufactured locally (the Supplementary Table S1 provide an overview per patient). The production processes of all radioligands were described previously [4,15,16,17,18]. In The Netherlands, the radiopharmaceuticals were synthesized according to Good Manufacturing Practice (GMP). In Germany, the production and administration were performed in accordance with the German Medical Products Act AMG §13.2b. PSMA-RLT was offered as a salvage therapy in accordance with paragraph 37, “Unproven Interventions in Clinical Practice” of the updated Declaration of Helsinki and in accordance with the local regulations as these patients refused chemo- or androgen deprivation therapy. Patients were treated in academic hospitals in either The Netherlands (Radboudumc Nijmegen) or Germany (Saarland University Hospital, Jena University Hospital or Magdeburg University Hospital). All patients were monitored in the outpatient clinic prior and after each therapeutic application and received regular assessment of blood values including hematology, chemistry and PSA levels. Clinically based treatment eligibility and discontinuation was decided by weekly tumor board discussion, attended by an oncologist, urologist and nuclear medicine physician. For data analyses, low volume or oligometastatic disease was defined as having a maximum of five metastases on PSMA-PET/CT, whereas those with more than five metastases were considered high volume [19].

### 2.3. Outcomes

The primary outcomes of this study were to determine the safety and the progression-free survival (PFS) of PSMA-RLT in patients with early-stage metastatic prostate cancer. Toxicity was scored according to the Common Terminology for Adverse Events (CTCAE) version 5.0. PFS was defined as the time interval between first PSMA-RLT injection till the earliest evidence of disease progression i.e., biochemical or clinical progression or death. According to PCWG3 criteria, PSA progression was defined as the first date a 25% PSA increase occurred from nadir [20]. If no decrease in PSA was observed, the first date of 25% increase from baseline was recorded. Clinical progression was defined following PCWG3 as the moment of: clinical deterioration (e.g., pain increment or start of new systematic treatment. Secondary endpoints were the PSA response defined as a ≥50% or ≥90% decrease in serum PSA from baseline and the time between initiation of new treatment or date of CRPC. CRPC was defined as a sequence of rising PSA levels with a minimum of 1-week interval (1.0 ng/mL initial value), with testosterone level of ≤50 ng/mL [20,21].

### 2.4. Statistical Analysis

Descriptive statistical methods were used to characterize the cohort groups. Survival analysis was performed by using the Kaplan-Meier statistics. All data was collected and managed using EPIC software and CastorEDC. Analyses were performed in SPSS version 27 software and in R statistics version 4.0.5. with ggplot2, dplyr, lifecycle packages.

### 2.5. Ethics

This study is in accordance with the Declaration of Helsinki. The study protocol for this retrospective analysis was approved by the Medical Review Ethics Committee Arnhem Nijmegen, The Netherlands CMO (2020-7526). Approval was received on the 23rd of December, 2020. All patients were well informed on the investigational product and its still experimental position in this early setting and provided written informed consent.

## 3. Results

### 3.1. Baseline Characteristics

**Figure 1:**
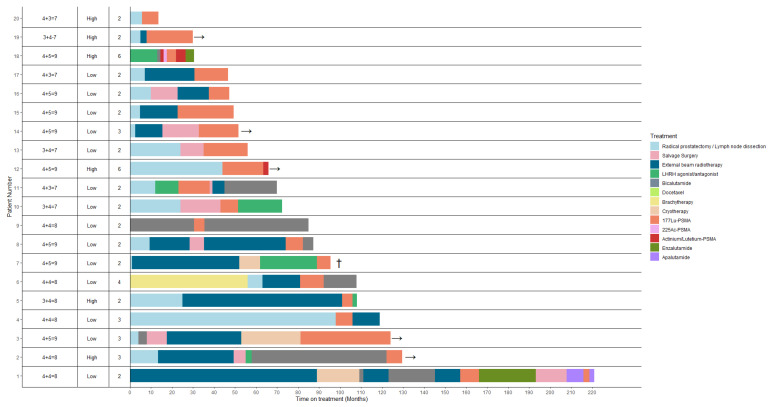
Swimmer plot illustrating the duration in months of each treatment per patient. Columns from left to right, initial Gleason Score; tumor volume at baseline (low volume was defined as having a maximum of 5 metastases); the amount of cycles each patient has received. Five patients have an ongoing response (indicated by the arrow), one patient has deceased (indicated by cross).

In total 20 prostate cancer with early phase metastasized patients prior to castrations resistant stage, and/or prior to hormonal therapy fulfilled the inclusion criteria of this study. The baseline characteristics of the patient cohort are described in Table 1. The median age of the patients was 69 years (interquartile range (IQR) 65.5–74), median PSA levels at the start of PSMA-RLT therapy was 5.9 µg/l (IQR 1.8–16.9). Fourteen patients had oligometastatic/low volume-disease on PSMA-PET/CT imaging, whereas six patients were considered high volume. All patients had lymph node metastases, whereas six patients (30%) had additional bone metastases. Two patients received both ^177^Lu-PSMA and ^225^Ac-PSMA (tandem treatment), and 18 patients received monotherapy ^177^Lu-PSMA. In these two patients, ^225^Ac was added to the regimen to provide stronger tumoricidal effects in at least two cycles. The median number of cycles was 2 (range 1–6) with a median of 6.2 GBq (IQR 5.4–7.9) ^177^Lu-PSMA per cycle. The swimmer plot provides a detailed overview of prior treatment lines and PSMA-RLT (Figure 1).

### 3.2. Toxicity

Overall, treatment injections of PSMA-RLT were well tolerated (Table 2). Six patients (30%) reported grade 1 xerostomia, which was deemed related to PSMA-RLT. Two of these patients received tandem therapy. One (5%) patient who received solely ^177^Lu-PSMA (cumulative activity of 12.4 GBq) developed grade 2 dry mouth and therefore stopped with PSMA-RLT. The xerostomia in these patients was transient and was not reported at a maximum of 12 months following treatment injections. Grade 1–2 treatment-related fatigue was observed in eight patients (40%), grade 1 nausea was observed in four (20%) patients. These adverse events were also transient. One (5%) patient developed grade 1 anemia likely related to PSMA-RLT. One (5%) patient developed grade 4 of thrombocytopenia and grade 3 anemia, which was likely related to the PSMA-RLT. For which he received blood transfusion. The patient was treated with four cycles of monotherapy ^177^Lu-PSMA, and thereafter with two cycles of tandem with both ^177^Lu- and ^225^Ac-PSMA. At the study cut-off, the adverse event has not been resolved.

### 3.3. Treatment Outcome

**Figure 2:**
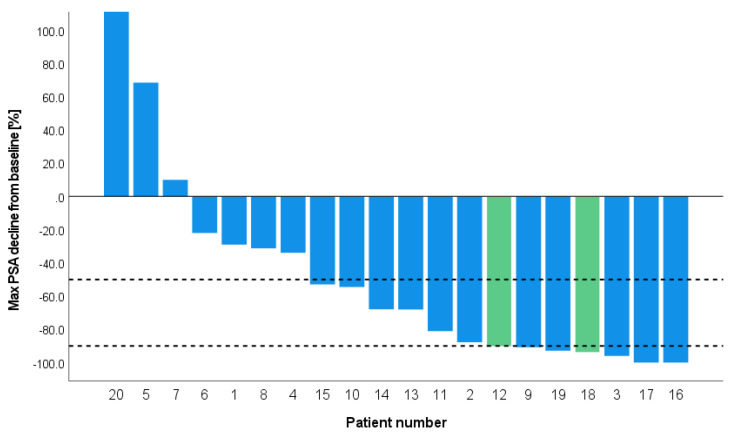
Waterfall plot of the individual changes of best PSA-response following PSMA−RLT. Blue bars indicate patients treated with ^177^Lu−PSMA and green bars with ^177^Lu−PSMA and ^225^Ac−PSMA. The two black lines represent ≥50% and ≥90% PSA decline. mHSPC = metastatic hormone sensitive prostate cancer; PSA = prostate specific antigen; PSMA = prostate specific membrane antigen; RLT = radioligand therapy.

**Figure 3:**
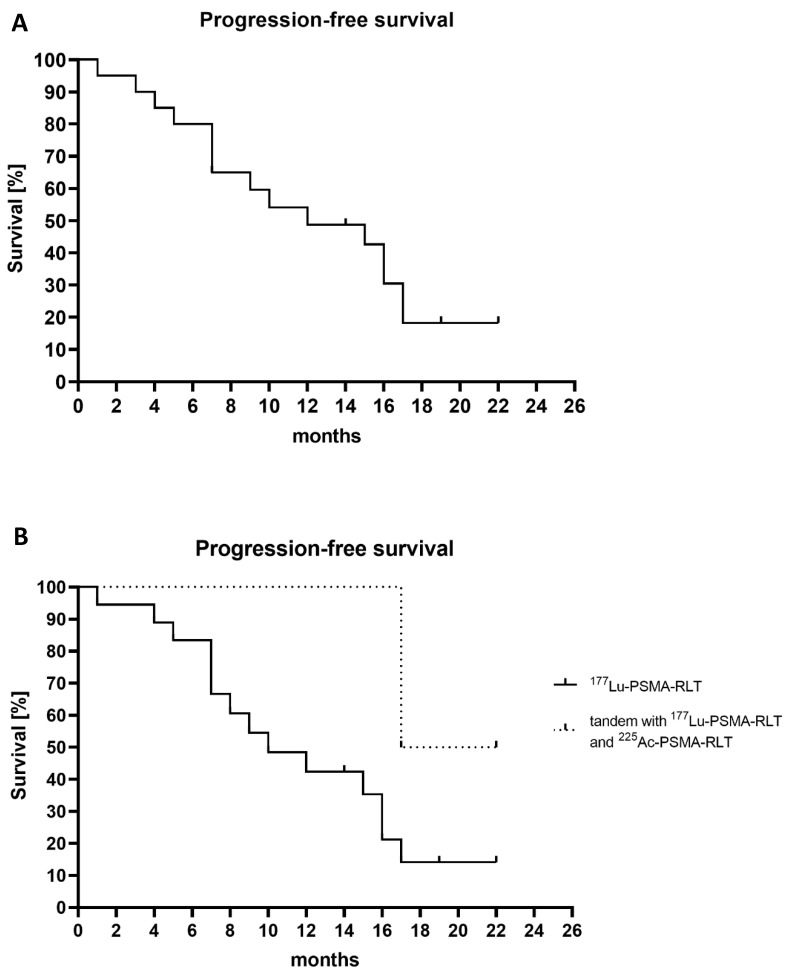
Survival analysis of progression-free survival in all early-stage prostate cancer patients. (**A**) The median PFS is 15 months in all mHSPC patients. (**B**) Stratified to ^177^Lu-PSMA (*n* = 18) vs. tandem of ^177^Lu-PSMA and ^225^Ac-PSMA (*n* = 2), the median PFS was 12 and 17 months respectively. mHSPC = metastatic hormone-sensitive prostate cancer; PFS = progression-free survival; PSA = prostate specific antigen; PSMA = prostate specific membrane antigen.

The median follow-up from the first cycle of PSMA-RLT was 20 (IQR 14.5–29.8) months. Seventeen patients (85%) had a PSA response, with 13 patients (65%) having a ≥50% PSA decline. Seven patients (35%) even had a PSA decline ≥90%. Both patients treated with a tandem of ^177^Lu and ^225^Ac-PSMA therapy showed a more than 90% decline in PSA. Figure 2 presents a waterfall plot of the best PSA response in all patients. Thirteen patients fit the definition of biochemical progression and two patients of clinical progression (one patient with pain increment and one patient started a new systemic treatment). Five patients have a stable disease and are still deferring a following treatment line (see Figure 1). The median PFS of all the patients was 12.0 (95% confidence interval [CI], 4.09–19.9) months (Figure 3A). The patients who were treated with only ^177^Lu-PSMA had a median PFS of 10 (95% CI,4.1–15.7) months (Figure 3B), whereas the two patients that additionally received ^225^Ac-PSMA (tandem) had a PFS of 17 and 22 months, respectively. Within the study window (12th of 2016 till June 1st of 2021), one patient developed CRPC nine months after the last cycle of PSMA-RLT and one patient deceased (cause of death was unrelated to PSMA-RLT).

## 4. Discussion

Only sparse data is available regarding the use of PSMA-RLT in hormone-sensitive prostate cancer patients. In this retrospective study, we evaluated the safety and efficacy of two different PSMA-RLT schemes (monotherapy of ^177^Lu-PSMA and ^177^Lu-PSMA in combination with ^225^Ac-PSMA) in 20 patients with early-stage metastatic prostate cancer patients, and generally prior to application of ADT, and/or even prior to hormonal therapy. Moreover, this is the first study including two patients with early-stage metastatic prostate cancer who were treated with tandem treatment. All patients in this study were either ineligible to the standard of care or refused any other conventional treatment due to unacceptable side effects (e.g., from ADT). Patients choose to receive PSMA-RLT after being well informed on PSMA-RLT and its experimental position in this early setting. During follow-up (median 20 months, IQR 14.5–29.8), we observed that PSMA-RLT with ^177^Lu-PSMA appeared to be safe with primarily low-grade transient toxicity. Moreover, a PSA response was observed in most patients along with an encouraging PFS.

PSMA-RLT was well tolerated in all patients. Within our study, there were no life-threatening treatment-related toxicities observed, besides, one patient who developed grade 4 bone marrow toxicity (anemia) for which he received blood transfusion. This patient was treated with four cycles of ^177^Lu-PSMA (cumulative activity of 27 GBq) and two cycles of tandem treatment with both ^177^Lu-PSMA (cumulative activity 14.5 GBq) and ^225^Ac-PSMA (cumulative activity 12.0 MBq). The other patients, had no or low-grade hematotoxicity, after a median follow-up of 20 months (range 7–51). We observed grade 1 xerostomia in six of 20 patients and grade 2 xerostomia in one patient. The dry mouth was generally transient, even the patient with grade 2 xerostomia did not report inconveniences regarding his saliva production one year after treatment. The low rate of adverse events was probably related to the lower amount of treatment activity applied and good organ reserve (e.g., bone marrow) of the patients in this study compared to patients reported in the VISION-trial [22]. Hence, the present cohort was in an early disease stage and received fewer (or no) systemic treatment lines compared to CRPC patients. Furthermore, it is also possible that most of our patients have not received the critical doses of RLT for organ-related toxicity, with a median cumulative total of 13.5 (IQR 9.0–20.7) GBq of ^177^Lu-PSMA. It was previously reported that the salivary glands, kidneys and bone marrow, receive a mean 0.39 ± 0.17 Gy/GBq, 0.49 ± 0.11 Gy/GBq, 0.017 ± 0.008 Gy/GBq of ^177^Lu-PSMA, respectively. Therefore, it has been suggested that up to 38 GBq of ^177^Lu-PSMA could be administered before clinically relevant organ-related toxicity is observed [5]. However, the application of higher activity of ^225^Ac-PSMA in early-stage patients requires careful consideration, particularly as the in vivo stability of ^225^Ac-PSMA radio-ligand complex is still unelucidated [23].

The first reports on ^177^Lu-PSMA in hormone-sensitive prostate cancer are showing promising efficacy [4,24,25]. In line with the earlier reports, we observed a ≥50% PSA decline in 65% of the patients. This is relatively higher than the published results for mCRPC patients following ^177^Lu-PSMA (~50%) [9,26,27]. Moreover, the median PFS was 12 months in the present study, whereas the PFS in mCRPC is around 9 months [3,28]. We postulate that this is related to patients in an earlier disease stage generally having a better-differentiated tumor subtype or a lower tumor burden and are thus more responsive to treatments. Although it is difficult to compare our small and heterogenous cohort to literature, the observed PFS of a median of 12 months is longer than the reported data of patients without treatment (median 5.6 months) [29]. Most of our patients (85%) had a complete/partial response or stable disease following PSMA-RLT and thus postponed the following treatment line. However, the present results should be interpreted with caution and more studies are needed in early-stage metastatic prostate cancer patients [4,5,8,30,31,32,33,34,35,36,37].

This is the first study to report on the tandem application of ^177^Lu-PSMA and ^225^Ac-PSMA in early-stage metastatic prostate cancer patients. The two patients treated with this regime showed ≥90% PSA decline and a PFS of ≥17 months, this is comparable with studies in mCRPC showing better response rates following ^225^Ac-PSMA [10,11,14]. Based on their physical properties, alpha emitters are considered to be more effective compared to beta emitters [38,39,40,41]. However, the mechanism of action is probably even more complex with secondary immune effects as reported for ^223^Ra [42]. Importantly, while alpha emitters may have higher efficacy, they may also be more toxic as mentioned above. Therefore, more studies are needed to evaluate radiolabeled α-emitters such as ^225^Ac-PSMA in clinical trials.

The study has several limitations. The retrospective nature of the data collection may have led to several forms of bias such as inherent reporting bias. The patient cohort is heterogeneous and, while all patients were considered hormone-sensitive as they were still responding to ADT, some did receive (novel) hormonal agents (e.g., anti-androgens or enzalutamide) or chemotherapy before PSMA-RLT. Therefore, some patients may not be considered truly hormone-sensitive or early-stage. Moreover, since these patients actively deferred from the standard of care (e.g., ADT, anti-androgens and chemotherapy), they are difficult to compare to the general patient cohort.

We believe this study offers informative data to colleagues working in the field of radioligand therapies on the long-term outcomes and safety of PSMA-RLT in an earlier stage of the disease. We therefore chose to release this manuscript. Properly powered randomized studies are on the way in a similar patient setting to truly evaluate the efficacy of PSMA-RLT early-stage cancer patients such as in hormone-sensitive setting (NCT04430192, NCT04443062, NCT04720157, NCT04343885, NCT04297410 & NCT05079698).

## 5. Conclusions

To conclude, in our cohort study of patients with early-stage (mainly hormone-sensitive) metastatic PSMA-RLT appeared safe during follow-up (median 20 months; IQR 14.5–29.8) with limited and mainly transient side effects. Importantly, PSMA-RLT showed encouraging efficacy for early-stage metastatic disease. The addition of ^225^Ac-PSMA to ^177^Lu-PSMA may result in better response rates but could result in high-grade toxicity and therefore restricted to specific patients. Prospective studies are awaited and should include long-term follow-up.

## Figures and Tables

**Table 1:** Baseline characteristics.

Characteristics	mHSPC Total (*n* = 20)	^a^ Tandem of ^177^Lu-PSMA and ^225^Ac-PSMA (*n* = 2)	^b 177^Lu-PSMA (*n* = 18)
Age, *median (IQR)* PSA doubling time >6 months, *n (%)*	69 (65.5–74) 5 (25%)	76 (72–80) 1 (50%)	68 (64.8–73.3) 4 (22.2%)
Previous treatments Radical Prostatectomy, *n(%)* PLND, *n(%)* External beam radiotherapy, *n (%)* Focal Cryotherapy, *n(%)* Hormonal therapy, *n (%)* ➢ LHRH agonist/antagonist ➢ Bicalutamide Previous chemotherapy (e.g., docetaxel), *n (%)*	15 (75%) 15 (75%) 15 (75%) 3 (15%) 8 (40%) 3 (15%) 7 (35%) 2 (10%)	1 (50%) 0 0 0 1 (50%) 1 (50%) 1 (50%) 1 (50%)	14 (77.8%) 15 (83.3%) 15 (83.3%) 3 (16.7%) 7 (38.9%) 2 (11.1%) 6 (33.3%) 1 (5.6%)
Site of disease on PSMA-PET/CT scan * Low volume disease, *n (%)* Hottest lesion, SUVmax, *median (IQR)* Lymph nodes, *n (%)* Bone, *n (%)* Visceral, *n* (%)	16 (80%) 28.8 (11.5–36.3) 20 (100%) 6 (30%) 1 (5%)	0 (0%) 49.1 (44.3–53.8) 2 (100%) 1 (50%) 1 (50%)	16 (88.9%) 20.4 (11.1–32.4) 18 (100%) 5 (27.8%) 0 (0%)
PSMA radioligand therapy Total number of PSMA-RLT cycles *median (range)* Administered activity of ^177^Lu (GBq), per cycle *median (IQR)* Administered activity of ^225^Ac (MBq) per cycle *median (IQR)*	2 (1–6) 6.2 (5.7–7.4) 4.2 (2.0–6.0)	6 (6–6) 7.2 (5.7–7.4) 4.2 (2.0–6.0)	2 (1–4) 6.1 (4.5–7.4) 0 (0–0)
Blood analysis Baseline Hemoglobin (mmol/L), *median (IQR)* Leukocytes (×10^9^/L), *median (IQR)* Thrombocytes (×10^9^/L), *median (IQR)* eGFR (mL/min), *median (IQR)* Creatinine (µmol/L), *median (IQR)* Alkaline phosphatase (U/L), *median (IQR)* Lactate dehydrogenase (U/L), *median (IQR)* PSA (µg/L), *median (IQR)*	8.9 (8.4–9.4) 5.7 (4.3–7.5) 232 (175–249) 79.9 (72–90) 81 (75–89) 63 (55.3–71.5) 196 (174–218) 5.9 (1.8–16.9)	9.5 (9.1–10) 6.1 (6–6.2) 204 (178–229) 77 (77–78) 85 (84–87) 85.0 (72–98) 204.5 (192–217) 146 (20–272)	8.8 (8.4–9.4) 5.3 (4.1–8.8) 238 (174–250) 84 (71–90) 80 (75–90) 59.5 (54.8–70) 195.5 (172–219) 5.1 (1.8–12.8)

^a^ Patients were included if they received ≥ 1 cycle of tandem therapy, ^b^ patients were solely treated with ^177^Lu−PSMA. * Low volume disease was defined as max 5 metastases. Data for the tandem patients are given in mean (range).

**Table 2:** Adverse events, possibly related to PSMA-RLT In 20 mHSPC patients.

	Grade 1	Grade 2	Grade ≥ 3
Xerostomia	6 (30%) *	1 (5%)	0
Fatigue	8 (40%) *	1 (5%)	0
Nausea	4 (20%)	0	0
Anemia	1 (5.0%)	0	1 (5%) *
Thrombocytopenia	0	0	1 (5%) *

Data is in *n*(%). * Indicates patients who were treated with both ^177^Lu-PSMA and ^225^Ac-PSMA (tandem).

## Data Availability

All patients characteristics were collected and recorded in a validated electronic case report form (https://www.castoredc.com/ (accessed on 14 June 2021)) and are available upon reasonable request to corresponding author.

## Supplementary Materials

The following supporting information can be downloaded at: https://www.mdpi.com/article/10.3390/cancers15010297/s1, Table S1: Administrated activity of 177Lu- and 225Ac-PSMA for each individual patient.

## References

[1] Siegel R.L., Miller K.D., Fuchs H.E., Jemal A. (2021). Cancer Statistics, 2021. CA Cancer J. Clin..

[2] Ahmadi H., Daneshmand S. (2013). Androgen deprivation therapy: Evidence-based management of side effects. BJU Int..

[3] Morris M.J., De Bono J.S., Chi K.N., Fizazi K., Herrmann K., Rahbar K., Tagawa S.T., Nordquist L.T., Vaishampayan N., El-Haddad G. (2021). Phase III study of lutetium-177-PSMA-617 in patients with metastatic castration-resistant prostate cancer (VISION). J. Clin. Oncol..

[4] Privé B.M., Peters S.M.B., Muselaers C.H.J., van Oort I.M., Janssen M.J.R., Sedelaar J.P.M., Konijnenberg M.W., Zámecnik P., Uijen M.J.M., Schilham M.G.M. (2021). Lutetium-177-PSMA-617 in low-volume hormone-sensitive metastatic prostate cancer: A prospective pilot study. Clin. Cancer Res..

[5] Peters S.M.B., Privé B.M., de Bakker M., de Lange F., Jentzen W., Eek A., Muselaers C.H.J., Mehra N., Witjes J.A., Gotthardt M. (2021). Intra-therapeutic dosimetry of [^177^Lu]Lu-PSMA-617 in low-volume hormone-sensitive metastatic prostate cancer patients and correlation with treatment outcome. Eur. J. Nucl. Med. Mol. Imaging.

[6] Privé B.M., Janssen M.J.R., van Oort I.M., Muselaers C.H.J., Jonker M.A., de Groot M., Mehra N., Verzijlbergen J.F., Scheenen T.W.J., Zámecnik P. (2020). Lutetium-177-PSMA-I&T as metastases directed therapy in oligometastatic hormone sensitive prostate cancer, a randomized controlled trial. BMC Cancer..

[7] Privé B.M., Janssen M.J.R., van Oort I.M., Muselaers C.H.J., Jonker M.A., van Gemert W.A., de Groot M., Westdorp H., Mehra N., Verzijlbergen J.F. (2021). Update to a randomized controlled trial of lutetium-177-PSMA in Oligo-metastatic hormone-sensitive prostate cancer: The BULLSEYE trial. Trials.

[8] Privé B.M., Slootbeek P.H.J., Laarhuis B.I., Naga S.P., van der Doelen M.J., van Kalmthout L.W.M., de Keizer B., Ezziddin S., Kratochwil C., Morgenstern A. (2021). Impact of DNA damage repair defects on response to PSMA radioligand therapy in metastatic castration-resistant prostate cancer. Prostate Cancer Prostatic Dis..

[9] Rahbar K., Ahmadzadehfar H., Kratochwil C., Haberkorn U., Schafers M., Essler M., Baum R.P., Kulkarni H.R., Schmidt M., Drzezga A. (2017). German multicenter study investigating ^177^Lu-PSMA-617 radioligand therapy in advanced prostate cancer patients. J. Nucl. Med. Off. Publ. Soc. Nucl. Med..

[10] Khreish F., Ebert N., Ries M., Maus S., Rosar F., Bohnenberger H., Stemler T., Saar M., Bartholomä M., Ezziddin S. (2020). ^225^Ac-PSMA-617/^177^Lu-PSMA-617 tandem therapy of metastatic castration-resistant prostate cancer: Pilot experience. Eur. J. Nucl. Med. Mol. Imaging.

[11] Sathekge M., Bruchertseifer F., Knoesen O., Reyneke F., Lawal I., Lengana T., Davis C., Mahapane J., Corbett C., Vorster M. (2019). Morgenstern A^225^Ac-PSMA-617 in chemotherapy-naive patients with advanced prostate cancer: A pilot study. Eur. J. Nucl. Med. Mol. Imaging.

[12] Sathekge M., Bruchertseifer F., Vorster M., Lawal I.O., Knoesen O., Mahapane J., Davis C., Reyneke F., Maes A., Kratochwil C. (2020). Predictors of overall and disease-free survival in metastatic castration-resistant prostate cancer patients receiving ^225^Ac-PSMA-617 radioligand therapy. J. Nucl. Med. Off. Publ. Soc. Nucl. Med..

[13] Van der Doelen M.J., Mehra N., van Oort I.M., Looijen-Salamon M.G., Janssen M.J.R., Custers J.A.E., Slootbeek P.H.J., Kroeze L.I., Bruchertseifer F., Morgenstern A. (2021). Clinical outcomes and molecular profiling of advanced metastatic castration-resistant prostate cancer patients treated with ^225^Ac-PSMA-617 targeted alpha-radiation therapy. Urol. Onco. Semin. Orig. Investig..

[14] Kratochwil C., Haberkorn U., Giesel F.L. (2020). ^225^Ac-PSMA-617 for therapy of prostate cancer. Semin. Nucl. Med..

[15] Cardinale J., Schafer M., Benesova M., Bauder-Wust U., Leotta K., Eder M., Neels O.C., Haberkorn U., Giesel F.L., Kopka K. (2017). Preclinical evaluation of ^18^F-PSMA-1007, a new prostate-specific membrane antigen ligand for prostate cancer imaging. J. Nucl. Med. Off. Publ. Soc. Nucl. Med..

[16] Fendler W.P., Calais J., Eiber M., Flavell R.R., Mishoe A., Feng F.Y., Nguyen H.G., Reiter R.E., Rettig M.B., Okamoto S. (2019). Assessment of ^68^Ga-PSMA-11 PET accuracy in localizing recurrent prostate cancer: A prospective single-arm clinical trial. JAMA Oncol..

[17] Kratochwil C., Bruchertseifer F., Giesel F.L., Weis M., Verburg F.A., Mottaghy F., Kopka K., Apostolidis C., Haberkorn U., Morgenstern A. (2016). ^225^Ac-PSMA-617 for PSMA-targeted alpha-radiation therapy of metastatic castration-resistant prostate cancer. J. Nucl. Med. Off. Publ. Soc. Nucl. Med..

[18] Kratochwil C., Giesel F.L., Stefanova M., Benesova M., Bronzel M., Afshar-Oromieh A., Mier W., Eder M., Kopka K., Haberkorn U. (2016). PSMA-targeted radionuclide therapy of metastatic castration-resistant prostate cancer with ^177^Lu-Labeled PSMA-617. J. Nucl. Med. Off. Publ. Soc. Nucl. Med..

[19] Aluwini S.S., Mehra N., Lolkema M.P., Oprea-Lager D.E., Yakar D., Stoevelaar H., van der Poel H., Busstra M., de Jong I.-J., Dutch Oligometastatic Prostate Cancer Working Group (2019). Oligometastatic prostate cancer: Results of a Dutch multidisciplinary consensus meeting. Eur. Urol. Oncol..

[20] Scher H.I., Morris M.J., Stadler W.M., Higano C., Basch E., Fizazi K., Antonarakis E.S., Beer T.M., Carducci M.A., Chi K.N. (2016). Trial design and objectives for castration-resistant prostate cancer: Updated recommendations from the prostate cancer clinical trials working group 3. J. Clin. Oncol. Off. J. Am. Soc. Clin. Oncol..

[21] Cornford P., van den Bergh R.C.N., Briers E., Van den Broeck T., Cumberbatch M.G., De Santis M., Santis M.D., Fanti S., Fossati N., Gandaglia G. (2021). EAU-EANM-ESTRO-ESUR-SIOG guidelines on prostate cancer. Part II—2020 Update: Treatment of Relapsing and Metastatic Prostate Cancer. Eur. Urol..

[22] Sartor O., de Bono J., Chi K.N., Fizazi K., Herrmann K., Rahbar K., Tagawa S.T., Nordquist L.T., Vaishampayan N., El-Haddad G. (2021). Lutetium-177–PSMA-617 for metastatic castration-resistant prostate cancer. N. Engl. J. Med..

[23] Thiele N.A., Wilson J.J. (2018). Actinium-225 for targeted α therapy: Coordination chemistry and current chelation approaches. Cancer Biother. Radiopharm..

[24] Satapathy S., Das N., Sood A., Singh S.K., Goyal S., Madan R., Mittal B.R. (2021). Short-course ^177^Lu-PSMA-617 radioligand therapy in high-volume metastatic hormone-sensitive prostate cancer: Time to take the leap?. Eur. Urol..

[25] Demirkol M.O., Kiremit M.C., Acar O., Falay O., Ucar B., Esen T. (2018). local salvage treatment of post-brachytherapy recurrent prostate cancer via theranostic application of PSMA-labeled lutetium-177. Clin. Genitourin. Cancer.

[26] Hofman M.S., Emmett L., Sandhu S., Iravani A., Joshua A.M., Goh J.C., Pattison D.A., Tan T.H., Kirkwood I.D., Ng S. (2021). [^177^Lu]Lu-PSMA-617 versus cabazitaxel in patients with metastatic castration-resistant prostate cancer (TheraP): A randomised, open-label, phase 2 trial. Lancet.

[27] Heck M.M., Tauber R., Schwaiger S., Retz M., D’Alessandria C., Maurer T., Gafita A., Wester H.J., Gschwend J.E., Weber W.A. (2019). Treatment outcome, toxicity, and predictive factors for radioligand therapy with ^177^Lu-PSMA-I&T in metastatic castration-resistant prostate cancer. Eur. Urol..

[28] Hofman M.S., Violet J., Hicks R.J., Ferdinandus J., Thang S.P., Akhurst T., Iravani A., Kong G., Ravi Kumar A., Murphy D.G. (2018). [^177^Lu]-PSMA-617 radionuclide treatment in patients with metastatic castration-resistant prostate cancer (LuPSMA trial): A single-centre, single-arm, phase 2 study. Lancet Oncol..

[29] Phillips R., Shi W.Y., Deek M., Radwan N., Lim S.J., Antonarakis E.S., Rowe S.P., Ross A.E., Gorin M.A., Deville C. (2020). Outcomes of observation vs stereotactic ablative radiation for oligometastatic prostate cancer: The ORIOLE phase 2 randomized clinical trial. JAMA Oncol..

[30] Iravani A., Violet J., Azad A., Hofman M.S. (2020). Lutetium-177 prostate-specific membrane antigen (PSMA) theranostics: Practical nuances and intricacies. Prostate Cancer Prostatic Dis..

[31] Violet J., Jackson P., Ferdinandus J., Sandhu S., Akhurst T., Iravani A., Kong G., Kumar A.R., Thang S.P., Eu P. (2019). Dosimetry of ^177^Lu-PSMA-617 in metastatic castration-resistant prostate cancer: Correlations between pretherapeutic imaging and whole-body tumor dosimetry with treatment outcomes. J. Nucl. Med.: Off. Publ. Soc. Nucl. Med..

[32] Gaertner F.C., Halabi K., Ahmadzadehfar H., Kurpig S., Eppard E., Kotsikopoulos C., Liakos N., Bundschuh R.A., Strunk H., Essler M. (2017). Uptake of PSMA-ligands in normal tissues is dependent on tumor load in patients with prostate cancer. Oncotarget.

[33] Edler von Eyben F., Singh A., Zhang J., Nipsch K., Meyrick D., Lenzo N., Kairemo K., Joensuu T., Virgolini I., Soydal C. (2019). ^177^Lu-PSMA radioligand therapy of predominant lymph node metastatic prostate cancer. Oncotarget.

[34] Paschalis A., Sheehan B., Riisnaes R., Rodrigues D.N., Gurel B., Bertan C., Ferreira A., Lambros M.B.K., Seed G., Yuan W. (2019). Prostate-specific membrane antigen heterogeneity and DNA repair defects in prostate cancer. Eur. Urol..

[35] Gafita A., Calais J., Grogan T.R., Hadaschik B., Wang H., Weber M., Sandhu S., Kratochwil C., Esfandiari R., Tauber R. (2021). Nomograms to predict outcomes after ^177^Lu-PSMA therapy in men with metastatic castration-resistant prostate cancer: An international, multicentre, retrospective study. Lancet Oncol..

[36] Peters S.M.B., Hofferber R., Privé B.M., de Bakker M., Gotthardt M., Janssen M., de Lange F., Muselaers C.H.J., Mehra N., Witjes J.A. (2021). [^68^Ga]Ga-PSMA-11 PET imaging as a predictor for absorbed doses in organs at risk and small lesions in [^177^Lu]Lu-PSMA-617 treatment. Eur. J. Nucl. Med. Mol. Imaging.

[37] Privé B.M., Derks Y.H.W., Rosar F., Franssen G.M., Peters S.M.B., Khreish F., Bartholomä M., Maus S., Gotthardt M., Laverman P. (2021). ^89^Zr-labeled PSMA ligands for pharmacokinetic PET imaging and dosimetry of PSMA-617 and PSMA-I&T: A preclinical evaluation and first in man. Eur. J. Nucl. Med. Mol. Imaging.

[38] Müller C., Umbricht C.A., Gracheva N., Tschan V.J., Pellegrini G., Bernhardt P., Zeevaart J.R., Köster U., Schibli R., van der Meulen N.P. (2019). Terbium-161 for PSMA-targeted radionuclide therapy of prostate cancer. Eur. J. Nucl. Med. Mol. Imaging.

[39] Müller C., Vermeulen C., Köster U., Johnston K., Türler A., Schibli R., Schibli R., van der Meulen N.P. (2016). Alpha-PET with terbium-149: Evidence and perspectives for radiotheragnostics. EJNMMI Radiopharm. Chem..

[40] Hindié E., Zanotti-Fregonara P., Quinto M.A., Morgat C., Champion C. (2016). Dose deposits from ^90^Y, ^177^Lu, ^111^In, and ^161^Tb in micrometastases of various sizes: Implications for radiopharmaceutical therapy. J. Nucl. Med..

[41] Uusijärvi H., Bernhardt P., Rösch F., Maecke H.R., Forssell-Aronsson E. (2006). Electron- and positron-emitting radiolanthanides for therapy: Aspects of dosimetry and production. J. Nucl. Med.: Off. Publ. Soc. Nucl. Med..

[42] Morris M.J., Corey E., Guise T.A., Gulley J.L., Kevin Kelly W., Quinn D.I., Quinn D.I., Scholz A., Sgouros G. (2019). Radium-223 mechanism of action: Implications for use in treatment combinations. Nat. Rev. Urol..

