# Supplementary material for: PSMA-RLT in Patients with Metastatic Hormone-Sensitive Prostate Cancer: A Retrospective Study
Source: Cancers (Basel). 2022 Dec 31;15(1):297. doi: 10.3390/cancers15010297 (PMC9818570; doi:10.3390/cancers15010297)
Supplement: Supplementary file 1 [file cancers-15-00297-s001.zip › cancers-2072637-supplementary.pdf]

Supplementary files

Supplementary Table S1
Administrated activity of ^{177}Lu- and ^{225}Ac-PSMA for each individual patient

**Cycles**	**1**		**2**		**3**		**4**		**5**		**6**	
**PSMA – RLT activity**	^{177}**Lu**	^{225}**Ac**	^{177}**Lu**	^{225}**Ac**	^{177}**Lu**	^{225}**Ac**	^{177}**Lu**	^{225}**Ac**	^{177}**Lu**	^{225}**Ac**	^{177}**Lu**	^{225}**Ac**
Patient 1	8.5		7.4									
Patient 2	7.4		7.4		7.3							
Patient 3	7.5		7.5		7.4							
Patient 4	7.4		6.9		6.9		7.0					
Patient 5	7.4		7.4									
Patient 6	3.1		6.1		6.2							
Patient 7	3.1		6									
Patient 8	3		6									
Patient 9	6.2		6.2									
Patient 10	3.1		6									
Patient 11	8.5		7.4									
Patient 12	6.6		5.7		7.4		7.3		7.2*	6.0*	7.3*	6.0*
Patient 13	3		5.9									
Patient 14	3		6		7.3							
Patient 15	3		6									
Patient 16	3.1		6									
Patient 17	3		6									
Patient 18	5.6	2.4	5.3	1.3		2.2	7.4		7.4		6.2*	6.1*
Patient 19	6.3		5.8		7.3							
Patient 20	3		4.9									

*Indicates cycles in which the radioligand label PSMA-I&T was utilized, for the remaining cycles PSMA-617 was applied.
